# Speed and Location Both Matter: Antigen Stimulus Dynamics Controls CAR-T Cell Response

**DOI:** 10.3389/fimmu.2021.748768

**Published:** 2021-10-08

**Authors:** Can Liu, Timothy Qi, J. Justin Milner, Yong Lu, Yanguang Cao

**Affiliations:** ^1^ Division of Pharmacotherapy and Experimental Therapeutics, School of Pharmacy, University of North Carolina at Chapel Hill, Chapel Hill, NC, United States; ^2^ Lineberger Comprehensive Cancer Center, School of Medicine, University of North Carolina at Chapel Hill, Chapel Hill, NC, United States; ^3^ Department of Microbiology and Immunology, Wake Forest School of Medicine, Winston-Salem, NC, United States

**Keywords:** CAR-T exhaustion, CAR-T activation, antigen presentation, discontinuity theory, solid tumor

## Abstract

Despite the success in B-cell malignancies, chimeric antigen receptor (CAR)-T cell therapies have not yet demonstrated consistent efficacy across all patients and tumor types, particularly against solid tumors. Higher rates of T cell exhaustion are associated with inferior clinical outcomes following CAR-T cell therapy, which is prevalent in solid tumors. T cell exhaustion may originate from persistent and chronic antigen stimulation by tumor cells that resist and/or evade T cell-mediated killing. We exploited CAR-T exhaustion with a classic negative feedback model (incoherent feedforward loop, IFFL) to investigate the balance between CAR-T cell activation and exhaustion under different antigen presentation dynamics. Built upon the experimental and clinical data, we hypothesize that the speed and anatomical location of antigenic stimulation are both crucial to CAR-T cell response. Chronic antigenic stimulation as well as the harsh tumor microenvironment present multiple barriers to CAR-T cell efficacy in solid tumors. Many therapeutic strategies are individually insufficient to improve of CAR-T responses against solid tumors, as they clear but one of the many barriers CAR-T cells face in solid tumors. A combination strategy targeting multiple barriers holds promise to improve CAR-T therapy in solid tumors.

## Introduction

Chimeric antigen receptor (CAR)-T cells are T cells that are genetically engineered to specifically recognize and lyse cells expressing their cognate antigen (1). Despite their rousing success in hematological malignancies, CAR-T therapies have not yet demonstrated reproducible efficacy across all patients and cancer types, particularly against solid tumors (2). CAR-T cells face multiple challenges in the solid tumor setting, including, but not limited to, the lack of truly tumor-specific antigens and the presence of hostile tumor immune microenvironments (3, 4). These challenges must be adequately addressed for CAR-T therapies to achieve success in solid tumors.

CAR-T cell functions are markedly impaired once they enter the exhaustion state. This cellular state is characterized by upregulation of inhibitory receptors (e.g., PD-1), specific epigenetic landscape and metabolism, and loss of effector function (5). The onset of exhaustion can lead to treatment resistance and/or cancer recurrence after CAR-T cell therapy. In addition to the tonic signaling that leads to variable levels of T cell exhaustion prior to antigen encounter, mounting evidence has shown that chronic and persistent antigen stimulation can induce T cell exhaustion, consistent with the basic working principle underlying the immune system known as the “discontinuity theory” (6–8). According to the “discontinuity theory”, the dynamics of antigen exposure play critical roles in activating T cells and their effector functions. This theory stems from the observation that the immune responses are more effectively induced by sudden changes in antigen concentration, whereas chronic and prolonged exposure to antigen leads to immune tolerance or cell exhaustion (9). Like endogenous T cells, CAR-T cells also drive the same types of programs seen in chronic and prolonged antigenic exposures that can result in exhaustion phenotypes (10–13).

This study employs a classic incoherent feedforward loop (IFFL) to revisit the “discontinuity theory” of immunity and extend it to CAR-T cell therapy. The impaired functionality of CAR-T cells upon chronic and prolonged stimulation by tumor antigens is likely associated with the presence of multiple regulatory feedback systems (14). A vital feature of the IFFL is that responses are proportional to the fold-change rather than the absolute change of a stimulus, much akin to Weber’s Law (15, 16). Here, we apply an IFFL to compare CAR-T cell activation and exhaustion under the distinct dynamics of antigen stimulus in hematological, as well as in solid tumor settings for exploring their distinct levels of response. The opportunities and limitations of prospective CAR engineering and therapeutic strategies are also evaluated to aid optimal design of solid tumor CAR-T cell therapeutics.

## Methods and Results

### The IFFL Recapitulates Negative Regulatory Mechanisms of CAR-T Cell Function

**Figure 1:**
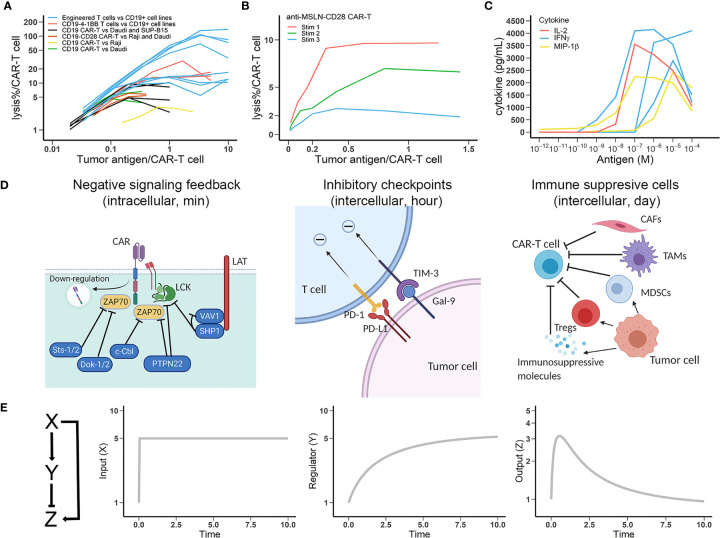
Multiple negative regulatory mechanisms that account for CAR-T cell exhaustion are captured by the incoherent feedforward loop (IFFL). **(A)** CAR-T cell lytic capacity (percentage of tumor cells lysed) at increasing densities of tumor antigens. Data are from multiple anti-CD19 CAR products. Data source: blue lines, engineered umbilical cord blood (UCB) T cells specifically killing CD19+ cell lines (19); red lines, cytotoxicity of CD19-4-1BBζ UCB T cells against Daudi, HPB, and K562-FCD19 cells (20); black lines, killing of Daudi and SUP-B15 cells by CD19ζ and CD19-CD28ζ CAR-T cells (21); brown lines, lytic activity of CD19-CD28ζ CAR against Raji and Daudi cells (22); yellow line, CAR-T cell cytotoxic activity against Raji cells (23); green lines, CD19ζ and CD19ζ-CD28 CAR-T cells lysed Daudi cells (24). Lines in the same color differ in the experimental conditions. **(B)** Lytic capacity (percentage of tumor cells lysed) of anti-MSLN-CD28-CAR-T upon repeated antigenic stimulation (25). **(C)** The secretion capacity of potent effector cytokines by CAR-T cells at varying antigen densities for 24 hrs (26). **(D)** Schematic view of negative regulatory mechanisms in CAR-T cell function. Left, negative feedbacks in intracellular CAR activation signaling; Middle, representative modes of intercellular inhibition by checkpoint molecules; Right, representative modes of intercellular inhibition mediated by tumor-resident cells, which can secrete soluble immunosuppressive molecules and express membrane-bound inhibitory molecules. **(E)** The IFFL with dynamic profiles of input, regulator, and output (27). The y-axe in figures is the fold-change from baselines (assumed to 1). Time is in arbitrary units in all plots. Using typical T cell regulation rate, the response time is in the range of minutes to hours.

Antigen engagement is a requisite step for CAR-T cell activation. Down-regulation of tumor antigen can lead to resistance to CAR-T cell therapy (17). However, continuously challenging CAR-T cells with antigen often results in dampened CAR-T function (18). Reduced CAR-T cell function at high target cells and tumor antigen densities have been shown in cell lysis assays and the per-unit diminution of CAR-T cell lytic capacity is evident as target density approaches an upper boundary (**Figure 1A**) (19–24). The loss of cytolytic function upon repeated antigen stimulation is also observed (**Figure 1B**) (25) and the dampened CAR-T function is even more apparent in cytokine secretion studies (**Figure 1C**) (26). This dampening highlights the critical role played by negative regulators of CAR-T cellular machinery (28).

Multiple mechanisms are possibly involved in CAR-T cell activation and exhaustion. The regulatory mechanisms of CAR-T adaptation, herein defined as the progressive loss of response to chronic and constant stimulus, are multifaceted and both CAR-T cell-intrinsic and -extrinsic. **Figure 1D** (left) shows that cell-intrinsic mechanisms can include surface CAR internalization and negative regulation of key downstream signaling elements. Downregulation of surface CAR has been broadly reported in many studies, especially after prolonged challenges with target cells (14, 29). CAR downregulation usually occurs immediately upon antigen binding, preventing CAR-T cell hyperactivation but also diminishing subsequent target-seeking ability. On the contrary, inhibiting lysosomal degradation markedly repressed CAR downregulation and promoted their long-term killing capacity (30). The degree of T cell adaptation caused by receptor (TCR or CAR) down-regulation is highly dependent on the antigen affinity (14). Antigens with stronger binding affinities result in higher and more durable TCR or CAR engagements, potentially leading to an elevated receptor down-regulation and T cell adaptation, similar as CAR-T cell adaptations at high antigen densities. Intracellularly, the regulators of intracellular signaling pathways include a diverse pool of tyrosine kinases, phosphatases, and transcriptional factors. For instance, upon antigen stimulation, LCK activates both the activating tyrosine kinase ZAP70 and its inhibitor SHP1 in what is referred to as the LCK-ZAP70-SHP1 loop, which leads to a rapid attenuation of T cell activation (31).

A second regulatory feedback mechanism is the upregulation of a repertoire of immune inhibitory receptors (**Figure 1D**, middle). Exhausted CAR-T cells express multiple inhibitory receptors (e.g., programmed cell death 1, PD1) that regulate effector function. These co-inhibitory receptors can be classified into various families on the basis of their structure and functions, wherein spatiotemporal expression curtail T cell functions in a highly context-dependent manner. Finally, the tumor microenvironment is rich with immune suppressive cells, including regulatory T cells (Tregs), myeloid-derived suppressor cells (MDSCs), tumor associated macrophages (TAMs), and cancer associated fibroblasts (CAFs) (**Figure 1D**, right). These extrinsic regulatory mechanisms can considerably diminish CAR activation and cause CAR-T cell exhaustion by expressing and secreting inhibitory factors (32, 33). Indeed, while these diverse cell-intrinsic and -extrinsic regulatory mechanisms ensure contextually-appropriate behavior for endogenous T cells, in the tumor they form the biological basis for CAR-T cell exhaustion.

Many studies have characterized the transcriptional and epigenetic alterations that define CAR-T cell exhaustion (34, 35). Here we focus on antigen stimulus dynamics and its influence on CAR-T activation using a reduced modeling framework – IFFL. We employ IFFL to interrogate intrinsic and extrinsic regulators of CAR-T cell exhaustion (27). We implemented the model in R, and the model equations and parameters are both provided in **Supplementary Material**. As depicted in **Figure 1E**, input (X), representative of tumor antigenic stimulation, can directly activate CAR-T cells (Z) while indirectly repressing CAR-T cell functions (Z) through a suite of multiple intermediate negative regulator (Y) as depicted in **Figure 1D**.

In the IFFL, when the input X is presented constantly, a transient increase of output Z is observed due to the temporal delay in repressor Y accumulation. As repressor Y accumulates, the output Z returns to baseline – perfectly, in the case of perfect adaptation. IFFL is a fundamental network motif broadly observed in biological sensory systems (e.g., vision and hearing) as well as gene regulation, in which an activator regulates both a gene and its repressor of the gene (36). Similar frameworks have been broadly applied to describe the regulatory nature of the immune system, particularly the T cell response (9). In the context of CAR-T cells, we apply IFFL to recapitulate antigen-dependent CAR-T cell activation and exhaustion.

### Antigen Presentation Dynamics Influences CAR-T Cell Response

**Figure 2:**
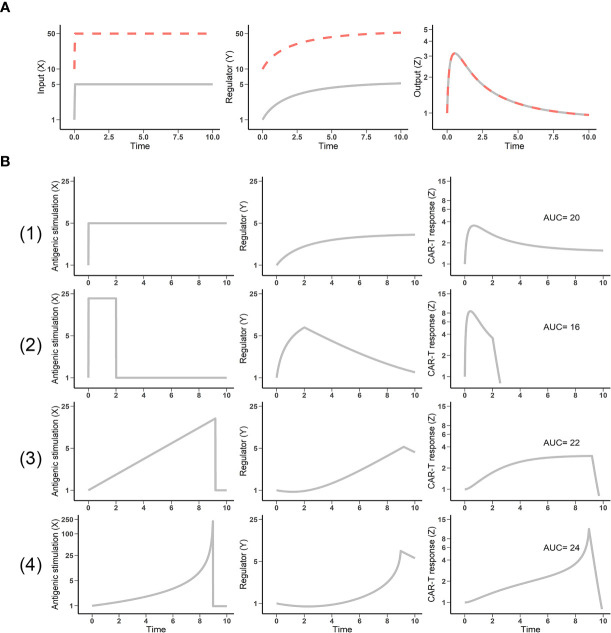
Fold-change detection in the incoherent feedforward loop (IFFL) reveals the impact of antigen presentation dynamics. **(A)** IFFL provides fold-change detection. Two inputs (gray and dashed red lines) of the same fold-change yield identical outputs, despite different baselines. **(B)** Antigen stimulus dynamics influences CAR-T cell response. Four patterns of antigen stimulus with equivalent area under the curve (AUC) were explored. (1) Constant appearance; (2) high but transient appearance; (3) linear appearance; and (4) exponential appearance of antigen (input X) yielded responses (output Z) with diverse amplitude, duration, and cumulative strength (AUC) profiles.

Mounting experimental evidence supports fold-detection as a feature of the immune system in pathological contexts such as cancer, viral infection, and allergies (8). One feature of the IFFL is fold-change detection, in which a response depends on the fold-change of the input, rather than on its absolute level. As shown in **Figure 2A**, two patterns of stimuli with five-fold increases from different baselines result in identical responses. This fold-change detection is the basis of “discontinuity theory” in the immune system. Importantly, this feature imbues CAR-T cells with high sensitivity to relative changes in antigen stimulus that can ultimately shape their response (9).

Antigen stimulus dynamics are a function of the interaction kinetics between CAR-T and tumor cell, which is extremely variable among cancer patients and contributes to the high variability in CAR-T cell proliferation and response. Within the IFFL, we simulated CAR-T cell responses by fixing total antigen stimulus exposures (equivalent total exposure of input X) and varying temporal patterns of presentation. Among these patterns, high but transient antigen stimulus results in the weakest CAR-T response (**Figure 2B**, second row). Exponentially increasing antigen stimulus yields the strongest response (**Figure 2B**, bottom row). T cells discriminate antigens through their dynamics. This theory has been supported by the experimental observations that the effector cells, including CAR-T cells, most effectively respond to antigenic threats with high changing rate (8, 14). CAR-T cell responses therefore depend not only upon the design of CAR and the T cells, but also upon the stimulatus patterns of their target antigen. The stimulus patterns of the target antigen to CAR-T cells is a function of tumor burden, anatomical site, antigen shedding level, and the heterogeneity in antigen-expression cells, among others. Overall, the presentation dynamics of target antigen can lead to highly variable CAR-T cell responses, which may result in high clinical variation and contribute to the discrepancy between hematological malignancies and solid tumor efficacy.

### CAR-T Cells Have Restricted Activation in Solid Tumors

**Figure 3:**
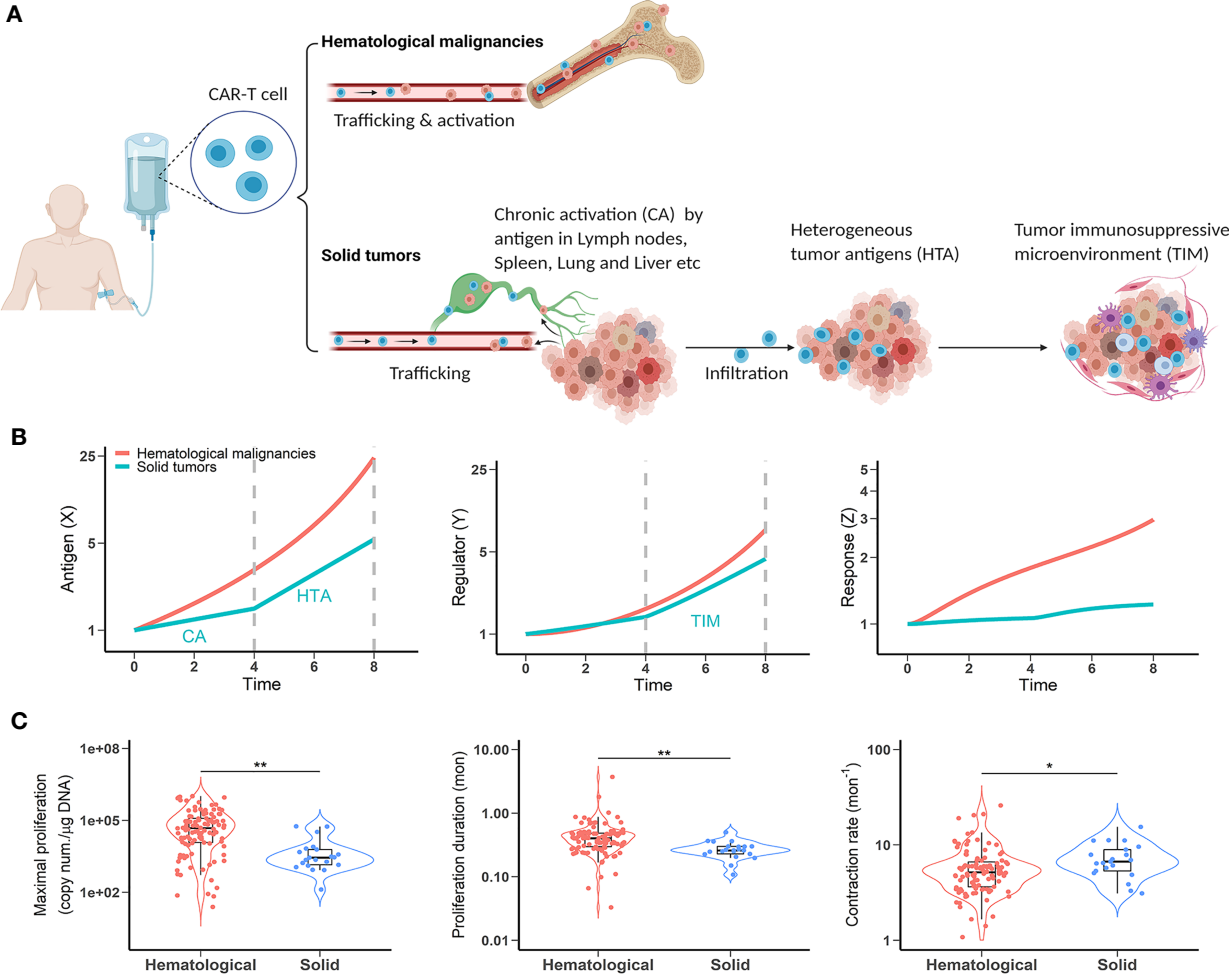
CAR-T cells have restricted activation in solid tumors. **(A)** A comparison of antigen exposure between hematological and solid malignancies and clinical observations. **(B)** The dynamic profiles of antigen stimulus (left), regulators (middle) and CAR-T response (right) in hematological and solid malignancies. When simulating mild chronic X to reflect chronic activation (CA) before tumor followed by exposure to highly heterogeneous antigen-expressing cells in the tumor (HTA), response (Z) is dampened by the TIM (part of Y). Vertical dashed lines indicate CAR-T cell distribution phases before (time 0-4) and after (time 4-8) tumor infiltration. **(C)** Clinical observations of CAR-T cellular kinetics in hematological malignancies and solid tumors. Left, maximal proliferation (the peak concentration of CAR copies, p < 0.001); Middle, proliferation duration (time from infusion to the peak concentration, p < 0.001); Right, contraction rate (slope of decline following initial peak, p < 0.05). The data points are from multiple CAR-T trials and according to our model-based cellular kinetic analysis (37).

Translating the efficacy of CAR-T cell therapy in hematological malignancies to solid tumors is a multifaceted challenge. CAR-T cell therapy has not yet shown reproducible clinical efficacy in solid tumors. Although anti-CD19 CAR-T cells undergo expansion post-infusion in most patients, CAR-T expansion has not been observed to the same extent in patients with solid tumors. We speculate it is because anti-CD19 CAR-T cells encounter their target antigen more readily than is the case in solid tumors. Due to their anatomical locations (most reside in the lymphoid tissues), hematological cancer cells encounter CAR-T cells and are expected to expose their target antigen to CAR-T cells more rapidly and frequently (**Figure 3A**). As a result, CAR-T cells are often extensively activated and exhibit considerable efficacy in hematological malignancies.

Unfortunately, CAR-T cells face additional challenges in solid tumor patients immediately after injection. First, there is a lack of highly tumor selective targets. Unlike the hematological antigens (e.g., CD19, CD22, and BCMA), most solid tumor-associated antigens are enriched in tumors but also shared by normal tissues. Whilst trafficking to tumor sites, on-target off-tumor expression may cause more chronic activation (CA) of CAR-T cells (**Figure 3A**). Solid tumors can also shed antigens into circulation, which may partially trigger CAR-T cells and cause chronic activation before tumor and exhaustion (38). Secondly, solid tumors consist of diverse cell populations with varying degrees of target expression. It is highly possible that CAR-T cells, after extravasation, have constrained access to highly antigen-expressing cells and are exposed to a diluted antigen pool (HTA, **Figure 3A**). As such, CAR-T cells are likely stimulated chronically in the tumor bed, resulting in a higher probability of exhaustion. Thirdly, the tumor microenvironment can drastically alter CAR-T cell function (TIM, **Figure 3A**). Compared to hematological malignancies, solid tumor microenvironments challenge CAR T-cells with hypoxic, acidic, and nutrient-poor conditions that contribute to altered metabolism and accelerated exhaustion. Furthermore, the abundance of immunosuppressive cells further impairs CAR-T function *via* presentation and secretion of immunosuppressive ligands and molecules.

We contextualize the model with these negative factors and evaluate their effects on CAR-T cell response. In addition to the diluted and chronic profiles of antigenic stimulation in solid tumors, we set a higher regulator (Y) production rate to reflect the immunosuppressive factors in the tumor microenvironment. As shown in **Figure 3B**, owing to the distinct antigenic presentation dynamics, CAR-T cells show a markedly dampened response in solid tumors compared to hematological malignancies.

We further verified our model prediction with clinical data. We surveyed the cellular kinetics of CAR-T cells in seven clinical trial cohorts of cancer patients (five hematological and two solid tumors) and extracted data including maximal proliferation (highest concentration of CAR copies in the blood), proliferation duration (time from injection to peak concentration), and contraction rate (slope of decline following the initial peak) across multiple CAR products and tumor types (37). These trials included 217 patients and 22 of which were from the solid tumor trials. Two solid tumors are glioblastoma and non-small cell lung cancer, treated with anti-EGFRvIII and anti-EGFR CAR-T cell therapies, respectively. Despite high inter-subject variabilities, we found that in solid tumors, CAR-T cells exhibited restricted proliferation potential (**Figure 3C**, left) and significantly shortened proliferation duration (**Figure 3C**, middle), whereas their contraction rates were significantly elevated (**Figure 3C**, right) compared to hematological tumors, consistent with our theoretical predictions in **Figure 3B**. These clinical observations support our model predictions to show CAR-T dampened activations in solid tumors. The diluted and chronic dynamic nature of antigen stimulus in solid tumors, coupled with the confluence of immunosuppressive factors, impose limited efficacy on CAR-T cell therapy in solid tumors.

### CAR Therapeutic Strategies Confer Differential Benefit to CAR-T Therapy

Multiple CAR engineering and therapeutic approaches are under active investigation to enhance CAR-T cell function *in vivo*. These strategies include combining CAR-T cell therapy with checkpoint blockade or other tumor immune modulators, cytokine-secreting “armored” CAR-T cells, CARs with OR logic gates, and regional injection of CAR-T cells (39, 40). Many of these approaches have shown promise for overcoming certain obstacles in solid tumors. We revisited these strategies and estimated their potentials and limitations for improving CAR-T cell responses against solid tumors under the “discontinuity theory.”

**Figure 4:**
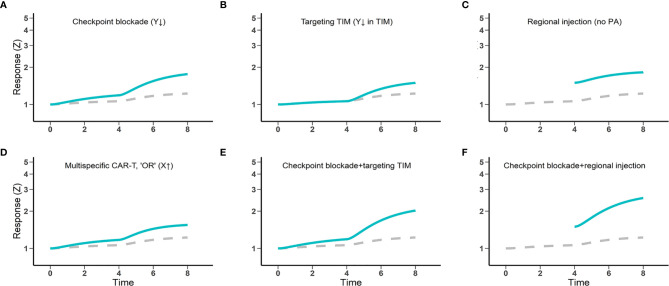
The potential and limitations of several engineering and therapeutic strategies for enhanced CAR-T cell efficacy against solid tumors. Gray dashed lines, CAR-T cell therapy control; Blue lines, CAR-T cell therapy combined with other strategies **(A, B, E, F)**, regionally administered CAR-T cells **(C, F)**, and multi-specific CAR-T cells **(D)**. Response (Z) generally indicates the magnitude of CAR-T cell activation and functions, including cytokine production potential and cytotoxic effects.

Immune checkpoint blockade can partially augment CAR-T cell therapy by counteracting the immune inhibitory receptors (41). In non-Hodgkin lymphomas, patients who initially failed to respond or relapsed to CD19 CAR-T therapy showed re-expanded CAR-T cells after a sequential treatment with the anti-PD1 inhibitor pembrolizumab (42, 43). This partial synergistic combination is also captured within “discontinuity theory” (**Figure 4A**). The magnitude of synergy depends on the fraction of exhausted CAR-T cells amenable to reinvigoration by checkpoint blockade, which can be highly variable in the clinic. It explains why checkpoint blockade does not consistently enhance CAR-T cell responses (44). Secondly, CAR-T cells must overcome the immunosuppressive microenvironments of solid tumors. Therapies that counteract immunosuppressive factors could be indispensable to achieving optimal effector function of CAR-T cells. Strategies such as targeting immunosuppressive cell types (Tregs, TAMs) or inhibiting immunosuppressive factors (e.g., TGF-β, IDO) have been preliminarily explored. These strategies can reprogram or normalize the tumor microenvironment to be more permissive to CAR-T cell effector functions (**Figure 4B**). Thirdly, intralesional injection has been employed to circumvent poor CAR-T cell trafficking and infiltration (45). Local CAR-T cell delivery in solid tumors, in addition to boosting CAR-T cell accumulation within tumors, may also partially avoid chronic activation before tumor by non-tumor antigens (**Figure 4C**). Currently, however, regional injections are primarily used for glioblastomas and malignancies with pleural or peritoneal spreading. Finally, multi-specific CAR with “OR” logic gates can activate CAR-T cells in the presence of either targeted antigen, partially overcoming heterogeneous expression of target antigens within the tumor (**Figure 4D**). Overall, our model corroborates the potential of each strategy to improve CAR-T cell responses in solid tumors, although no individual strategy alone seems capable of boosting CAR-T response levels to those comparable with what we observed in hematological tumors.

The journey of CAR-T cells driving to solid tumors is riddled with roadblocks. Together, these roadblocks impinge CAR-T cells’ overall trafficking into solid tumors in a potentially redundant manner, such that individual roadblocks may act as independently rate-limiting steps even when others are cleared. Based on our predictions, combination strategies that overcome multiple roadblocks may be a promising path toward achieving robust benefit of CAR-T cell therapy in solid tumors (**Figures 4E, F**).

## Discussion

CAR-T cell therapy has become a revolutionary treatment in light of their remarkable efficacy in hematologic malignancies. Unfortunately, in patients with solid tumors, consistent responses to CAR-T cell therapy are still limited. The use of an antigen-binding antibody fragment in CARs enables CAR-T cells to recognize and kill tumor cells independently of the major histocompatibility complex (MHC). Incorporating a costimulatory receptor domain (CD28 or 4-1BB) into the CAR allows CAR-T cells to respond independently of costimulatory signaling. However, the conventional design of CARs does not preclude the onset of multiple co-inhibitory signaling mechanisms, nor does it avoid the harshly immunosuppressive tumor microenvironment. As a result, CAR T cell exhaustion is augmented and efficacy is diminished against solid tumors. Removal of one of the genes encoding these co-inhibitory receptors, such as PD-1 (*PDCD1*), has shown the potential to improve antitumor immunity (46, 47).

It is well documented that T cell dysfunction and exhaustion are prevalent in solid tumors. Like physiological T cells, CAR-T cells are susceptible to chronic antigenic stimulation and exhaustion. In this study, we applied an IFFL to the “discontinuity theory” of the immune system to assess the dynamics of antigen presentation and its consequence on CAR-T cell response and exhaustion. Our findings highlighted that chronic activation (CA) before tumor and diluted (HTA) antigenic stimulation, as well as the tumor immunosuppressive microenvironment (TIM), can significantly restrain CAR-T cell responses against solid tumors. The antigen presentation rate (the number of antigens presented to each CAR-T cell per unit of time) and the location of malignant cells both critically affect the exhaustion and response of CAR-T cells.

Although insufficient antigen access leads to CAR-T cells’ poor functions in solid tumors, excess antigenic stimulation or too high tumor burdens may also result in dysfunctions of CAR-T cells. There is likely a goldilocks of antigen stimulation for CAR-T cells, where CAR-T cells should be appropriately stimulated to function but are not overwhelmed by the amount of tumor antigens. Resistance to CD19 CAR T-cell often develops in patients with high tumor burdens (48). Excess co-stimulation or CAR with very high target affinity does not always translate to better clinical outcomes (18, 49). Consistent with this observation, increasing CAR affinity is known to promote T cell exhaustion and decrease the generation of memory-phenotype cells (50). The model suggests that the proper magnitudes and temporal dynamics of antigen presentation are essential for an efficient CAR-T cell therapy, which yields multiple implications for patient selection and CAR designs.

Multiple therapeutic approaches to enhance CAR-T cells efficacy against solid tumors are under active investigation. The therapeutic promise of these approaches has been preliminarily observed in certain solid malignancies (51, 52). However, translating the dramatic efficacy of CAR-T cells in hematological settings into solid tumor contexts remains challenging. We contextualized these therapeutic approaches with an IFFL and investigated their potential to enhance CAR-T cell responses. Interestingly, we found each of these approaches was individually effective but insufficient to boost CAR-T responses against solid tumors, as they clear but one of the many roadblocks CAR-T cells face on their journey toward efficacy in solid tumors. For instance, combining checkpoint blockades with CAR-T cell therapies could partially reverse CAR-T cell exhaustion (41). Still, such a combination has not significantly benefited CAR-T cell functions in solid tumors yet (53). Checkpoint blockades may release the brakes from suppressed CAR-T cells, but such a combination is insufficient to induce T cells infiltration and reverse the immunosuppressive factors in solid tumors. Therefore, as our model implicates, studies combining multiple mitigating strategies may be necessary to result in T cell infiltration, activation, and effector function in complex solid tumors (54).

Our reductionist modeling approach for CAR-T cell activation is primarily a proof-of-concept, which comes with limitations. CAR-T cell activation and exhaustion are highly complex biological processes that involve many phenotypic, transcriptional, and epigenetic alterations. Our model only considers the antigen-dependent CAR-T cell simulation. Antigen-independent CAR-T cell activation (55) and high levels of tonic signaling due to CAR aggregation (56, 57) can also induce CAR-T cell exhaustion. This further obfuscates the balance between CAR-T cell activation and exhaustion. Our model does not explicitly integrate these regulatory factors, considering these immunosuppressive factors may occur on different spatiotemporal scales. Although it can improve model fidelity, integrating these factors into the model requires more quantitative investigations of these regulatory factors, which are unfortunately still emerging. Tonic CAR signaling, triggered by clustering of CAR, can induce early exhaustion of CAR T cells. Different costimulatory domains may confer different sensitivity to exhaustion induced by persistence CAR signaling; for instance, CD28 appears to augment, while 4-1BB ameliorates, exhaustion induced by CAR clustering. In addition to antigen dynamics, this balance is also a function of CAR affinity for antigens, antigen density on target cells, and the nature of intracellular costimulatory domains, each of which can enhance or reduce CAR-T cell activation. More theoretical and experimental investigations into these aspects are warranted to score a clinically meaningful strike against solid tumors.

## Author Contributions

Conceptualization: YC. Investigation: CL. Analysis: CL. Writing-original draft preparation: YC and CL. Writing-review and editing: YC, CL, TQ, JM, and YL. Supervision: YC. Funding acquisition: YC. All authors have read and agreed to the published version of the manuscript.

## Funding

This study was funded by National Institute of Health (GM119661).

## Conflict of Interest

TQ owns equity in Humanigen, a biopharmaceutical company.

The remaining authors declare that the research was conducted in the absence of any commercial or financial relationships that could be constructed as a potential conflict of interest.

## Publisher’s Note

All claims expressed in this article are solely those of the authors and do not necessarily represent those of their affiliated organizations, or those of the publisher, the editors and the reviewers. Any product that may be evaluated in this article, or claim that may be made by its manufacturer, is not guaranteed or endorsed by the publisher.
