# Supplementary material for: Speed and Location Both Matter: Antigen Stimulus Dynamics Controls CAR-T Cell Response
Source: Front Immunol. 2021 Oct 8;12:748768. doi: 10.3389/fimmu.2021.748768 (PMC8531752; doi:10.3389/fimmu.2021.748768)
Supplement: Supplementary file 1 [file DataSheet_1.docx]

Representative R code for simulations in Fig 1E and Fig 3B

Dynamic equations (35) for Y and Z are:

dY/dt =β_1_X-α_1_Y

dZ/dt= β_2_X/Y- α_2_Z

library(RxODE)

library(dplyr)

# Figure 1E

ode =" d/dt(X)= 0

d/dt(Y)= beta1*X-alpha1*Y

d/dt(Z)= (beta2*X)/Y-alpha2*Z"

model = RxODE(model = ode)

params = c(alpha1=0.25, alpha2=3, beta1=0.28, beta2=3)

inits = c(1,1,1)

events = eventTable() %>%

add.dosing(dose = 4, start.time = 0, dosing.to = 1) %>%

add.sampling(seq(0,10,by=0.01))

simulation = model$solve(params, events, inits)

# blood Fig 3B

ode = " d/dt(X)= (X/2.9)^1.4

d/dt(Y)= beta1*X-alpha1*Y

d/dt(Z)= (beta2*X)/Y-alpha2*Z "

model = RxODE(model = ode)

params = c(alpha1=0.28, alpha2=3, beta1=0.28, beta2=3)

inits = c(1,1,1)

events = eventTable() %>%

add.sampling(seq(0,8,by=0.01))

simulation = model$solve(params, events, inits)

# Solid tumor Fig 3B

ode =" if (t < 4) {a<-0.1} else {a <- 0.32}

d/dt(X)= a*X

d/dt(Y)= beta1*X-alpha1*Y

d/dt(Z)= (beta2*X)/Y-alpha2*Z "

model = RxODE(model = ode)

params = c(alpha1=0.28, alpha2=3, beta1=0.35, beta2=3)

inits = c(1,1,1)

events = eventTable() %>%

add.sampling(seq(0,8,by=0.01))

simulation = model$solve(params, events, inits)
